# Addressing the sexual and reproductive health of women with Bipolar Disease

**DOI:** 10.1192/j.eurpsy.2022.2235

**Published:** 2022-09-01

**Authors:** A. Vieira, F. Ramalheira, I. Caldas, I. Vidó

**Affiliations:** 1 Centro Hospitalar Psiquiátrico de Lisboa, Ccsmo, Lisbon, Portugal; 2 Centro hospitalar Psiquiátrico de Lisboa, Serviço De Electroconvulsoterapia, Lisboa, Portugal; 3 Centro Hospitalar Psiquiátrico de Lisboa, Psiquiatria Geral, Lisboa, Portugal

**Keywords:** bipolar disorder, women, Sexual and Reproductive Health

## Abstract

**Introduction:**

Bipolar disorder (BD) is a severe mental illness (SMI) with an estimated lifetime prevalence of around 1%, starting in young adulthood and progressing with acute episodes. Although there is no significant prevalence difference between the sexes, the course of the disorder may be more problematic in women, due to hormonal and reproductive factors. Moreover, hypersexuality and impulsive sexual behaviour can manifest as part of a manic or hypomanic episodes, with devastating effects on the physical and emotional health of these patients.

**Objectives:**

To highlight the pertinent issues related to sexual and reproductive health of women with BD.

**Methods:**

A non systematic review of the literature from the last 10 years was carried out using the electronic databases, Pubmed and Google Scholar. The literature search was confined to papers written in English. The keywords ‘sexual health’, ‘reproductive health’, were combined with ‘bipolar disorder’ and ‘women’.

**Results:**

The literature points to an increased incidence of unsafe sexual practices (unprotected sex, multiple sexual partners, trading sex) as well as poor reproductive and sexual health (increased risk of sexually transmitted diseases, high risk of unwanted pregnancies and abortions, low use of contraceptives, menstrual and fertility problems). Female patients with BD are also more likely to report history of sexual abuse.

**Conclusions:**

Attention and counseling regarding effective contraception, planning a pregnancy and risk of sexually transmitted diseases, among others, should be an integral part of health care received by all women with bipolar disorder.

**Disclosure:**

No significant relationships.

